# Laser polymerized photonic wire bonds approach 1 Tbit/s data rates

**DOI:** 10.1038/s41377-020-0292-1

**Published:** 2020-04-27

**Authors:** Saulius Juodkazis

**Affiliations:** 1 Nanotechnology facility at Optical Sciences Centre and ARC Training Centre in Surface Engineering for Advanced Materials (SEAM), Swinburne University of Technology, John st., Melbourne, 3122 VIC Australia; 2 Tokyo Tech World Research Hub Initiative (WRHI), Tokyo Institute of Technology, Tokyo, 152-8550 Japan

**Keywords:** Lithography, Physics

Microelectronics has solved the challenge of packaging different functional elements with integrated chips (ICs) in modern computing and communication by wire bonding. Miniaturization was a trend guided by the requirements for faster, more portable and less expensive (smaller amount of materials) solutions, where wire bonding evolved to accommodate increasingly more complex 3D architectures of chips and printed circuit boards.

Even faster and more robust computing and communication can be provided by using photons rather than electrons. This was one of the underlying reasons for the fast growth of optical fiber communication, which is especially efficient over large ~1000 km distances. Data transfer in microelectronics also increasingly benefits from optical interconnects, which, however, have scaling challenges at small IC dimensions.

The overall trend of optical fibers prevailing over copper wire for long-distance data transfer follows the miniaturization trend, and now, a new capability has been demonstrated on the microscale^1^. Photonic wire bonding^1,2^ solves the same problem as wire bonding in microelectronics, but for optical communication on the level of chip-to-chip interconnects. In a recent study^1^, a new milestone for data transfer over a 75 km channel at a rate of ~0.8 Tbit/s was demonstrated for Si photonic transmitters co-packaged with 1.5 μm InGaAsP lasers using photonic wire bonding. Several breakthroughs were combined to achieve the record high speed of data transfer. Optical insertion losses of only 0.7 dB were demonstrated. In addition, the required photonic wire tapering for single mode fiber operation/filtering was seamlessly achieved by direct laser writing. Most importantly, photonic wire bonding solves the stringent requirements for optical alignment between components and opens the possibility for automated packaging solutions. Si photonic platforms based on well-established complementary metal-oxide-semiconductor technologies can now be combined with other light sources and nonlinear optical components based on other platforms using photonic wire bonding. This makes hybrid photonic chip modules possible^1^. It can be seen as a manifestation of the evolving trend in modern technology where complexity develops along with miniaturization, which has been the guiding principle established in previous decades of microelectronics.

Ideally, photonic wire bonds should have the lowest possible losses, similar to gold (copper) wires in microelectronics. The demonstrated 0.7-dB optical insertion losses are on par with those in 1 km of an optical single mode fiber (0.2 dB/km). The flexibility of 3D direct free-form writing of photonic wire bonds using Nanoscribe IP-Dip organic–inorganic resist holds promise for further densification of photonic wire bonds up to 100 per millimeter of chip edge (with a 10-μm separation rather than the used 25 μm). A sub-100 nm precision in 3D positioning of the focal spot in liquid resist and 3D polymerization along the required trajectory following the designed pattern could be achieved in a fully automated mode of operation^1^.

**Fig. 1:**
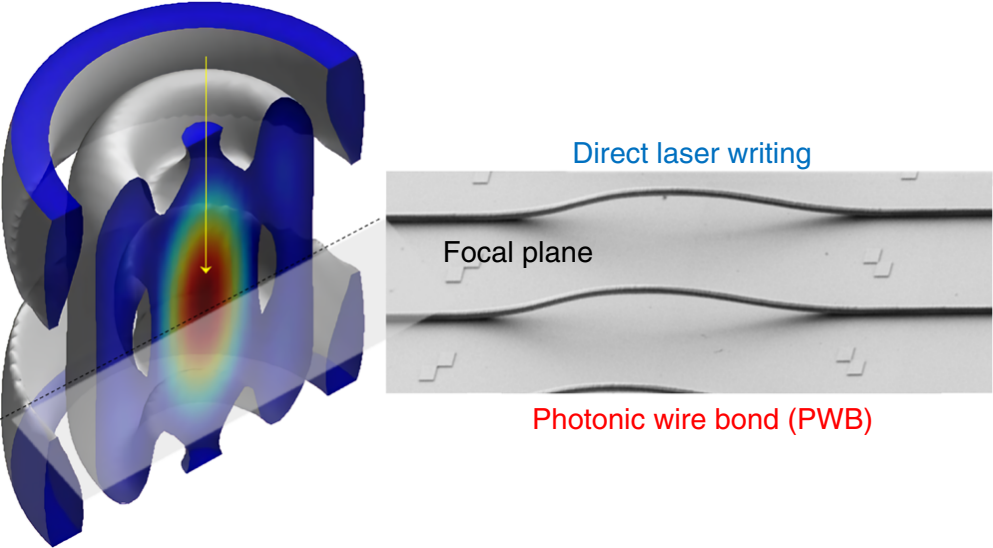
Photonic wire bonds (PWBs) are polymerized by direct laser writing using ultrashort laser pulses at a high repetition rate under tight focusing conditions. The focal volume has cross sections comparable to the wavelength of light of ~1 μm (see the 3D intensity cross section)

Direct laser writing under tight focusing (Fig. 1) was used to polymerize photonic wire bonds in a negative-tone liquid organic–inorganic resist/resin^1^. This method has an inherent subwavelength resolution for the 780-nm laser writer used due to the threshold effect of polymerization^3^. The high tens-of-megahertz laser repetition rate facilitated thermal polymerization, which created a more uniformly polymerized solid polymer phase as well as a smoother surface of the photonic wire bond. It is important to minimize structural nonuniformity and mass density fluctuations in the photonic wire bond since they cause Rayleigh scattering. In optical fibers, this scattering defines the minimum achievable optical losses. The telecom wavelength of 1.5 μm is at the exact minimum of the intersection between the Rayleigh scattering and overtone of the OH absorption band. Since the organic part of the resist and photoinitiator have overtones of their main IR absorption bands at near-IR 1–2 μm wavelengths^3^ for oxygen–carbon (methyl CH_3_, methylene CH_2_, methine CH), oxygen–hydrogen (hydroxyl –OH) and other groups, precise control of the resist composition becomes important. The overtone frequency  f = (1 − (*v* + 1)*x*)*vω* depends on the vibrational quantum number *v* = 1, 2, …, *n*, the anharmonicity constant *x*, and the frequency of bond vibration *ω*^4^ and contributes to the overall optical losses. The composition of organic–inorganic resists/resins can be optimized for a particular application by using wavelength-specific photoinitiators, abandoning them entirely^5^, or tailoring the exposure to the IR absorption bands of a polymer for crosslinking^6^. The strategy of minimizing absorption and scattering implemented in optical fibers is expected to guide future development of new materials for lossless photonic wire bonds.

Further possible advancements in the fabrication throughput and accuracy of photonic wire bonds is expected with the improvement of laser beam scanning via a combination of mechanical stages and galvano scanners delivering stitchless and arbitrary-size write fields^7^. In combination with the recent development of burst-mode ultra-short lasers, the thermal protocol of ablation^8^ or polymerization can be effectively tuned to obtain the highest efficiency of material processing. Direct laser writing of micro-optical elements and photonic wire bonds has become a promising branch of 3D printing with unique scaling flexibility that covers nano-to-macro sizes from tens of nanometer to millimeters using the same approach. This mesoscale capability is a particular virtue of laser writing that is useful for the fabrication of complex 3D structures.
